# Editorial

**Published:** 1877-11

**Authors:** 


### Editorial.

Advertising—Professional and Commercial.—Not long since two advertising pamphlets were extensively circulated throughout this city, largely filled with certificates of certain physicians of the city of New York, attesting the virtues of the Hunyadi Jànos, and the Apollinaris mineral waters. The majority of the names signed to the certificates appear in both of the pamphlets. To establish the identity of the persons, the certificates are preceded by elaborate headings, which narrate what chairs those making them, occupy in a particular college—the hospitals in which they hold appointments—and of one, we are even favored with his office address. The certificates are so much alike that the doctors could have exchanged signatures without doing each other injustice; or they might have written, as one did, "I fully concur in the above." A careful perusal of these pamphlets engenders the doubt whether the advertisements are designed chiefly for the benefit of the venders of these remarkable waters, or for that of certain doctors in New York city, who fill chairs in a college named—who are connected with certain hospitals, and who may be consulted by the afflicted at a widely advertised number and avenue. Immediately after these pamphlets had been disseminated through the mails, an abbreviation of the certificates, with the names of those making them, appeared in displayed letters in the most conspicuous place of the leading daily papers of the city of Chicago: and these advertisements have been reissued from day to day to the present time. The action of these physicians, if not less defensible, would be less noticed, if analysis of the waters revealed in them any unusual properties. The principal ingredients of the Hunyadi János water seem to be the sulphate of magnesia, and the sulphate and bicarbonate of soda; a simple saline laxative, such as may be obtained at every drug-store, however unpretending; or from the saddle bags of any backwoods' doctor. We never before have known so many eminent names invoked, for so simple a purpose, as to prescribe a "dose of salts." Truly a mountain has labored, and less than a mouse is produced.

We write both in sorrow and in anger. These gentlemen, standing in the medical profession among the first in America, owe an apology for this act; they should publicly make such penitential confession, as may prevent this disgrace from ever being quoted as a precedent. It is a palpable violation of the spirit: if not of the letter, of the code; and the code should be observed by members eminent in the profession, if not by others. If these certificates had appeared over the autographs of so many designing charlatans, there would have been a manifest fitness in the whole transaction. The city of New York is one of the centres of medical education in this country; and these saline professors are enrolled among its teachers; and this act of theirs is certainly a most reprehensible example to set before the pupils under their instruction, as well as to the entire profession and public. It is a satisfaction to us that we at no time have had occasion to blush for any such flagrant violation of professional propriety in the West.

